# Environmental enrichment and the sensory brain: the role of enrichment in remediating brain injury

**DOI:** 10.3389/fnsys.2014.00156

**Published:** 2014-09-02

**Authors:** Dasuni S. Alwis, Ramesh Rajan

**Affiliations:** Department of Physiology, Monash UniversityClayton, VIC, Australia

**Keywords:** EE, sensory cortices, traumatic brain injury, neuronal excitability

## Abstract

The brain's life-long capacity for experience-dependent plasticity allows adaptation to new environments or to changes in the environment, and to changes in internal brain states such as occurs in brain damage. Since the initial discovery by Hebb (1947) that environmental enrichment (EE) was able to confer improvements in cognitive behavior, EE has been investigated as a powerful form of experience-dependent plasticity. Animal studies have shown that exposure to EE results in a number of molecular and morphological alterations, which are thought to underpin changes in neuronal function and ultimately, behavior. These consequences of EE make it ideally suited for investigation into its use as a potential therapy after neurological disorders, such as traumatic brain injury (TBI). In this review, we aim to first briefly discuss the effects of EE on behavior and neuronal function, followed by a review of the underlying molecular and structural changes that account for EE-dependent plasticity in the normal (uninjured) adult brain. We then extend this review to specifically address the role of EE in the treatment of experimental TBI, where we will discuss the demonstrated sensorimotor and cognitive benefits associated with exposure to EE, and their possible mechanisms. Finally, we will explore the use of EE-based rehabilitation in the treatment of human TBI patients, highlighting the remaining questions regarding the effects of EE.

Experience-dependent plasticity encompasses a vast number of paradigms that range from deprivation to alterations and enrichments in the environment, and has been investigated in great detail across development through to adulthood (see reviews by Hubel and Wiesel, 1970; Hubel, 1978; Kaas, 1991; Klintsova and Greenough, 1999; Sur and Leamey, 2001; De Villers-Sidani and Merzenich, 2011; Bengoetxea et al., 2012). For the purposes of the present review, we chose to focus on plasticity conferred by global changes to the environment, termed environmental enrichment (EE). We will focus only on the changes evoked by this manipulation when applied in adulthood as our final aim is to demonstrate that it represents an exciting potential therapy in adult traumatic brain injury (TBI). As we shall review, EE alters neuronal function through a range of morphological and molecular interactions, which lead to alterations in sensorimotor and cognitive behavior. These changes make EE an ideal candidate in the treatment of TBI. To lead to this thesis, we first commence with a review of EE's capacity to evoke plasticity in the uninjured brain, to provide the context in which we will cast the role of EE in TBI. We focus here on EE-induced changes in sensory cortices due to the demonstrated effects of TBI on altering neuronal function in sensory cortices (Hall and Lifshitz, 2010; Ding et al., 2011; Alwis et al., 2012; Johnstone et al., 2013); it is also our view that changes in neuronal activity in sensory cortices after injury must underlie a significant portion of the persistent cognitive deficits found in TBI (Caeyenberghs et al., 2009; Davis and Dean, 2010; Lew et al., 2010; Folmer et al., 2011). The review of EE effects in the normal brain is also necessary to understand the mechanisms whereby EE produces changes in neuronal function in the normal brain, before we can begin to hypothesize about how EE exerts its beneficial effects after brain injury. Finally, we discuss the current literature regarding the use of EE as a potential therapy post-TBI, in animal studies with induced TBI, and in studies of human rehabilitation after injury.

## What is EE?

EE refers to an experimental paradigm in which laboratory animals are housed in an environment allowing cognitive, motor and sensory stimulation at levels much greater than those which occur under standard laboratory housing conditions (Hebb, 1947, 1949; Van Praag et al., 2000). Early studies in animals have shown that the enhanced stimulation from EE produces many remarkable benefits at anatomical, molecular and behavioral levels (Hebb, 1947; Bennett et al., 1969; Diamond et al., 1972, 1976; Greenough and Volkmar, 1973; Torasdotter et al., 1998), with numerous studies following on from this work to further characterize the effects of EE (see reviews by Van Praag et al., 2000; Nithianantharajah and Hannan, 2006). In EE, the housing environment is modified by providing a larger enclosure, natural bedding and a variety of novel objects, in the expectation that this will promote greater physical activity in exploration and interaction with a novel and complex environment (Benaroya-Milshtein et al., 2004; Zebunke et al., 2013). Social enrichment in the EE environment, involving housing animals with multiple cagemates to encourage complex social interactions (Rosenzweig et al., 1978; Mesa-Gresa et al., 2013), is also believed to contribute to an enhanced sensorimotor and cognitive experience. Enhanced physical activity and enhanced social interaction each provide benefits to the brain; physical activity on its own improves cognitive performance in parallel with a range of neural changes including enhanced neurogenesis and increased levels of neurotrophic growth factors and increased neurotransmitter subunit expression (Van Praag et al., 1999; Farmer et al., 2004; Erickson et al., 2011), while social enrichment on its own has been shown to result in an increase in brain weight (Rosenzweig et al., 1978). When the two are combined in an appropriately enriched environment, a much more extensive set of cerebral changes occurs (Rosenzweig et al., 1978; Johansson and Ohlsson, 1996; Sozda et al., 2010).

The combination of social, physical and cognitive stimulation is most often used in studies of EE and we term this “generic” EE, wherein the whole environment is non-selectively enriched. However, in some instances, in what we term “specific” EE, enrichment has been targeted to affect a specific system, e.g., auditory-specific enrichment (Engineer et al., 2004; Percaccio et al., 2005, 2007; Jakkamsetti et al., 2012) consisting of components of generic EE in combination with systems designed to produce a variety of salient sounds; or tactile-specific enrichment (Bourgeon et al., 2004; Xerri et al., 2005) where rats were raised in an environment consisting of objects with various textures. Differences in generic and specific EE will be highlighted further, in the context of EE effects on neuronal function in the cortex.

## Beneficial effects of EE on behavior

EE exposure results in a range of sensorimotor and cognitive benefits in laboratory animals, which we only briefly summarize as these have been well reviewed elsewhere (Van Praag et al., 2000; Nithianantharajah and Hannan, 2006; Simpson and Kelly, 2011). In normal animals, EE significantly improves spatial and non-spatial learning and memory, novel object discrimination, increases the speed of spatial learning and enhances spatial searching strategies (Van Praag et al., 2000; Schrijver et al., 2002; Nithianantharajah and Hannan, 2006; Kulesskaya et al., 2011; Vedovelli et al., 2011; Leger et al., 2012). EE appears to decrease anxiety, as evidenced in a variety of tests (Fernandez-Teruel et al., 2002; Larsson et al., 2002; Galani et al., 2007; Harati et al., 2013). EE also improves task-learning, and recent and remote memory retrieval (Harati et al., 2013), likely due to a greater ability to consolidate and retain information because of social enrichment (Gardner et al., 1975). However, effects are not all positive and studies have shown both increases and decreases in aggressive social behavior after EE (Abou-Ismail, 2011; Workman et al., 2011; McQuaid et al., 2012; Mesa-Gresa et al., 2013), possibly due to factors such as differences in EE housing conditions, strain differences, and experimental design.

In a similar vein, the consensus (Nithianantharajah and Hannan, 2006; Kazlauckas et al., 2011; Landers et al., 2011) is that EE encourages activity and exploratory behavior though there are some inconsistencies: some studies show increased activity in novel environments (Benaroya-Milshtein et al., 2004), and others show faster habituation and less activity (Zimmermann et al., 2001; Schrijver et al., 2002; Elliott and Grunberg, 2005), when compared with animals housed in standard or impoverished environments (Varty et al., 2000; Zimmermann et al., 2001). Recently, Zebunke et al. (2013) showed a decrease in general activity during an open field test, with an increase in duration of exploration of novel objects by pigs exposed to cognitive enrichment. Similarly, Mesa-Gresa et al. (2013) also found that EE rats exhibited longer durations of novel object exploration, while Schrijver et al. (2002) found an increase in activity in a light/dark box in EE rats. Bruel-Jungerman et al. (2005) have also reported that EE animals were capable of retaining memory during a novel object recognition test for up to 48 h after initial exposure, despite a lower object exploration time during the learning phase of the test.

Among the most robust of findings is that EE and sensory training/learning improves stimulus discrimination (Gibson, 1953; Kendrick et al., 1992; Recanzone et al., 1993). Mandairon et al. (2006a,b) have shown that olfactory enrichment results in an improved ability to discriminate between odor pairs, likely due to changes in neuronal response properties (Buonviso and Chaput, 2000; Fletcher and Wilson, 2003). Similarly, EE enhances spatial discrimination of sound source, with faster reaction times and improved discrimination accuracy (Cai et al., 2009). Bourgeon et al. (2004) reported that while EE housing did not affect an animal's tactile ability to discriminate between textured surfaces, enriched animals did learn to perform the discrimination task faster. The changes in behavior reported above must occur as a consequence of the effects of EE on neuronal function, which in turn, occur as a result of the various molecular and morphological changes mediated by EE.

## Neuronal functional changes associated with exposure to EE

The EE-induced changes in behavior can be linked to specific changes in neuronal functionality. This has been studied in best detail for behavior associated with hippocampal function (Van Praag et al., 2000; Eckert and Abraham, 2013) and we briefly describe these as a prelude to describing the changes seen in adult sensory cortices, the particular brain regions of interest here in the context of our over-arching thesis (Alwis et al., 2012) that many persistent cognitive and motor deficits in TBI have sensory deficits as an underlying cause. The studies discussed below have used electrophysiological techniques such as *in vivo* and *in vitro* intra and extracellular recordings to specifically investigate EE-induced changes in neuronal function.

EE-induced improvements in hippocampal-dependent memory function have been linked to experience-dependent changes in hippocampal synaptic strength (Kempermann et al., 1997; Schrijver et al., 2002; Vedovelli et al., 2011), with reports of increases in excitatory post-synaptic potential (EPSP) amplitudes and evoked population spikes in rats exposed to generic EE, both in *in vivo* studies (Sharp et al., 1985; Irvine and Abraham, 2005; Irvine et al., 2006) and in *in vitro* studies of slices from the dentate gyrus (Green and Greenough, 1986; Foster et al., 1996) or the CA3-CA1 pathway (Foster and Dumas, 2001; Malik and Chattarji, 2012). The enhanced synaptic efficacy in dentate gyrus appears likely to act through AMPA and NMDA receptor mediated mechanisms (Foster et al., 1996). Interestingly, these increases did not outlast the termination of EE housing (Green and Greenough, 1986), even though EE-induced changes in behavior and morphology persist after discontinuation of EE (Camel et al., 1986; Cheng et al., 2012), suggesting that information stored in the dentate gyrus may be related to more transient behavioral effects of EE. However, there is also contradiction in studies of EE-induced long-term hippocampal plasticity. Eckert and Abraham (2010) reported that long-term exposure to EE did not result in enhanced synaptic transmission in the hippocampus, both *in vivo* and *in vitro*, suggesting that the variability in these studies may have be due to different EE paradigms, or to homeostatic mechanisms to re-establish normal synaptic transmission (Turrigiano, 1999, 2008). Foster and colleagues (Foster et al., 1996; Foster and Dumas, 2001) demonstrated that EE housing inhibits LTP induction in the perforant pathway, suggesting that both experience-dependent synaptic plasticity and LTP expression share similar yet-unknown underlying mechanisms. Conversely, increased LTP expression has been reported after EE exposure (Duffy et al., 2001; Artola et al., 2006; Eckert and Abraham, 2010; Malik and Chattarji, 2012). One resolution for these effects, other than differences in the EE conditions, is that LTP induction after EE may be differentially regulated in different regions of the hippocampus.

It is worth noting here that short-term plasticity in the hippocampus has not been shown to be affected by EE (Foster et al., 1996; Foster and Dumas, 2001; Eckert and Abraham, 2010; Malik and Chattarji, 2012).

In contrast to the hippocampus, little is known about EE-induced changes in neuronal functionality in cortex. What changes there are in cortical neuronal function have mainly been examined at the level of the sensory cortices and we discuss these studies in detail below. EE-induced changes in neuronal function in the normal (uninjured) sensory cortices are particularly salient to our thesis and may provide us with insights into the role of EE on neuronal function after brain injury, of which nothing is known as yet.

### EE and sensory cortices

The effects of EE have been studied most extensively in auditory cortex, in some detail in somatosensory cortex, and only to a limited degree in visual cortex.

In auditory cortex, the effect of EE has been studied at levels ranging from brain slices through to extracellular recordings from neurons in anaesthetized animals. In the investigation of the effects of EE on the auditory cortex, studies have used enriched environments that include specific auditory enrichment in the form of playback of various sounds within the housing environment (Engineer et al., 2004; Percaccio et al., 2005, 2007; Nichols et al., 2007). Many studies report effects that mirror those seen in the hippocampus, of increased synaptic efficiency. Thus, auditory cortex slices show that specific EE induces an increase in excitatory post-synaptic current (EPSC) amplitudes, coupled with a decrease in current rise-times in supragranular cortical layers and no changes in infragranular layer V (Nichols et al., 2007). *In vivo* recordings from the anaesthetized rat, primarily from Layers 4/5 of adult primary auditory cortex after specific auditory EE, have demonstrated an increase in cortical responsiveness (both spontaneous and stimulus-evoked), decreased response latencies, and an increase in frequency selectivity (Engineer et al., 2004; Percaccio et al., 2005; Cai et al., 2009). Percaccio et al. (2005, 2007) also found that EE increased paired pulse depression (PPD) in the rat auditory cortex, indicating an increased probability of synaptic transmitter release and thus, enhanced synaptic transmission. Other studies in auditory cortex found EE could cause reorganization of the cortical tonotopic map (Norena et al., 2006; Pienkowski and Eggermont, 2009; Zhou et al., 2011; Kim and Bao, 2013), and alterations in stimulus frequency selectivity over either a range of frequencies (Zhou et al., 2011) or for frequencies specific to those used as a part of the enrichment condition (Norena et al., 2006; Pienkowski and Eggermont, 2009).

The effects of auditory enrichment are not restricted to primary auditory cortex, and Jakkamsetti et al. (2012) have reported that responses in posterior auditory field (PAF) are also increased when compared with animals housed in standard environments. These increased firing rates were accompanied by decreases in response latency and duration, and a reduction in receptive field size, as seen in primary auditory cortex (Engineer et al., 2004; Zhou et al., 2011; Jakkamsetti et al., 2012).

Unlike the above-noted reports, some studies do not report increased neuronal responsiveness and sharper frequency tuning after exposure to auditory enrichment (Condon and Weinberger, 1991; Bao et al., 2003). Instead, these studies found that a repeated auditory stimulus decreased responsiveness to frequencies used in the stimulus (Condon and Weinberger, 1991), and exposure to noise burst trains produced broadly tuned receptive fields (Bao et al., 2003). Percaccio et al. (2007) have suggested that a critical variable in eliciting EE effects in auditory cortex is the nature of the enrichment, i.e., how engaging or complex the stimuli are. This would explain the increased neuronal responsiveness reported by Percaccio et al. (2007) in rats receiving even passive exposure to specific auditory EE, which included situation-dependent stimuli from the environment and from cagemates, as opposed to simple, less behaviorally relevant stimuli.

Similar to studies in auditory cortex, generic EE (i.e., non-specific enrichment) results in reorganized cortical topographic maps, decreased receptive field sizes, increased response selectivity and increased sensory evoked potentials in the somatosensory cortex (Xerri et al., 1996; Coq and Xerri, 1998; Polley et al., 2004; Devonshire et al., 2010). One interesting effect demonstrated here is that EE effects on receptive field sizes and responses to stimulation of the main topographic input to the neurons may be laminar selective (an effect that does not appear to have been explored in auditory cortex). Thus, in the rodent barrel cortex that receives tactile input from the mystacial whiskers, EE caused a decrease in receptive field size and in neuronal responses evoked by stimulation of the “Principal Whisker” (the topographically matched whisker providing the main input to a group of neurons in the barrel cortex) in supragranular cortical Layers 2/3, but there were no changes in response strength or receptive field size in input Layer 4 (Polley et al., 2004). It is worth noting that Guic et al. (2008) found that EE caused an increase in cortical representational area in Layer 4. However, these effects were seen after stimulation of only a few selective whiskers, while other whiskers were trimmed whereas Polley et al. (2004) used a non-deprived paradigm where all whiskers remained untrimmed. These different effects are likely a reflection of variations in experimental design of the EE conditions, consistent with the conclusions drawn from studies in auditory cortex that the nature of EE conditions influences neuronal outcomes.

These studies, where the emphasis was on measuring receptive field sizes and responses only to simple input from the main topographically-matched region of the body, indicated laminar specificity of effects. However, when neuronal encoding of sophisticated sensory input is the metric, the effects of generic EE occur globally across all cortical layers. Thus, in our own studies, 8–10 weeks of EE exposure increased neuronal firing rates globally across all layers 2–5 of the rat barrel cortex, and did so in response to both simple stimuli and a variety of complex, naturalistic stimuli (Alwis and Rajan, 2013). It is interesting to note that these effects occur even to the complex stimuli as our previous work on TBI (Alwis et al., 2012) had suggested that the complex stimuli may engage a diversity of intra-cortical processing mechanisms not seen with the simple stimuli. These effects occurred without any change in response latency, suggesting that the effects were specific to cortex and not due to changes at lower levels of the somatic pathways to cortex.

Although not often studied on its own in somatosensory cortex, recently EE has been combined with another manipulation that induces experience-dependent plasticity in barrel cortex, viz. whisker trimming and/or stimulation (Armstrong-James et al., 1992; Diamond et al., 1993, 1994; Rema et al., 2006; Guic et al., 2008; Megevand et al., 2009). Here the picture is rather murky, with one study suggesting that EE operates through different mechanisms than other plasticity mechanisms in barrel cortex, but another suggesting that it operates through the same mechanisms. The first seems to apply in the case of whisker trimming: when whisker pairing (all whiskers on one side of the face trimmed except for a pair of adjacent whiskers) is coupled with short (15 h) generic EE exposure, there is an accentuation of the effects induced by whisker trimming alone: a faster shift of receptive field bias toward the untrimmed whiskers, stronger evoked responses to the intact paired whisker than to deprived whiskers, and increased spontaneous activity in supra-granular and granular layers (Rema et al., 2006). In contrast EE may operate through the same mechanisms as some other plasticity cases. Thus, a short duration of whisker stimulation at a frequency often used during exploratory whisking behavior increases stimulus evoked potentials in both supra-granular and granular barrel cortex layers (Megevand et al., 2009)—but, addition of EE to the whisker stimulation paradigm does *not* further potentiate responses (Megevand et al., 2009).

Finally, only a limited number of studies have examined the effects of EE in the normal visual cortex. In area 17 of the adult visual cortex, similar to effects seen in the auditory cortex, generic EE results in sharper bandwidths in orientation tuned cells, increased neuronal responses to light stimuli, increased visual acuity, as well as increased stimulus contrast and temporal selectivity (Beaulieu and Cynader, 1990a,b; Mainardi et al., 2010). In addition to these effects in normal adult visual cortex, studies of EE-induced plasticity in the adult visual cortex have also examined effects in the context of monocular deprivation (MD) and amblyopia. MD during developmentally critical periods induces a shift in ocular dominance (OD) so that more neurons respond to stimulation of the open eye (Frenkel and Bear, 2004; Mrsic-Flogel et al., 2007). Such plasticity is normally not seen when MD is started in adulthood, but EE housing for 3 weeks re-activates cortical plasticity in supragranular layers of adult visual cortex such that OD changes are possible again and visual evoked potentials (VEPs) elicited by stimulation of the deprived eye are greatly depressed (Baroncelli et al., 2010b). In amblyopia, individual components of EE such as motor and visual stimulation, as well as the combination of these components, also recover visual acuity and restore OD plasticity and binocularity in supragranular layers of adult visual cortex (Sale et al., 2007; Baroncelli et al., 2012; Tognini et al., 2012).

Taken together, these studies of EE effects in sensory cortices show that generic EE is potent at producing many changes in neuronal responses, such as stronger responses and greater stimulus selectivity; that the receptive field effects may be laminar-selective and depend on the type of enrichment but that effects on more sophisticated neuronal processing, particularly of naturalistic stimuli that mimic everyday events, occur across all cortical laminae; and finally, that EE may operate independent of some other mechanisms of cortical plasticity.

## Mechanisms underlying EE-induced changes in neuronal function

**Figure 1:**
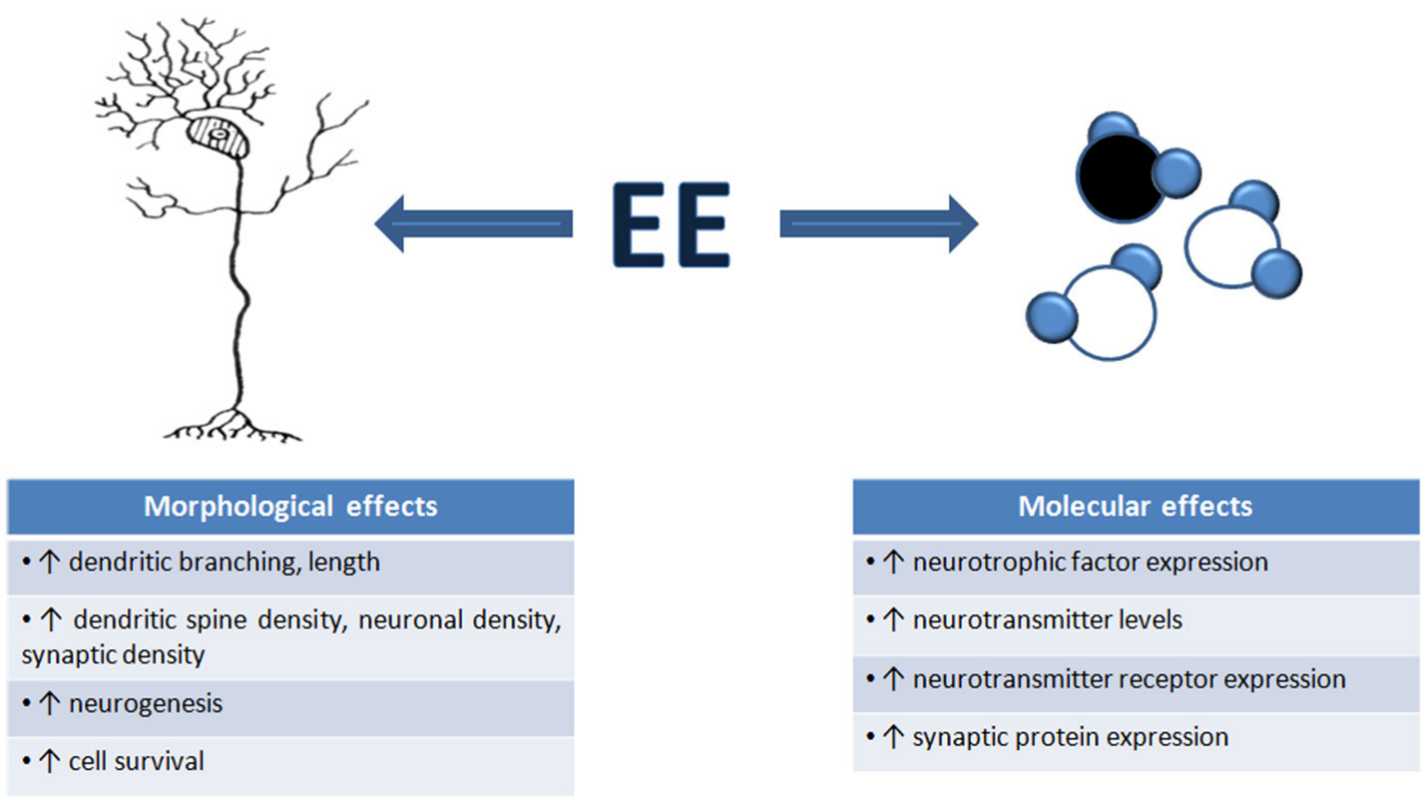
**Environmental enrichment induces morphological and molecular changes in the brain**. An overview of the number of structural and molecular mechanisms that contribute to the changes in neuronal function, and ultimately, changes in behavior, seen after EE exposure. These mechanisms are thought to underlie EE-induced neural plasticity.

There are numerous well-documented structural and biochemical consequences of EE which may underlie the effects of EE on neuronal function. We broadly review these changes with EE (summarized in Figure 1). It must be noted that in most cases, we do not know how these structural and molecular changes contribute to EE-induced changes in neuronal function: to date, there has been very limited attempt only to directly manipulate these fine-scale changes to determine to what extent they cause EE-related changes in neuronal functionality.

### Morphological changes

It was very early recognized that exposure to complex, enriched environments causes gross morphological changes in an overall increase in brain weight, particularly in cortical and hippocampal weight and thickness (Bennett et al., 1969; Walsh et al., 1969; Diamond et al., 1972, 1976). The factors contributing to these gross morphological changes include increased neuronal density and size, increased dendritic branching and length, increased dendritic spine density, and increased turnover in pyramidal and stellate cells (Holloway, 1966; Diamond et al., 1967; Volkmar and Greenough, 1972; Globus et al., 1973; Greenough and Volkmar, 1973; Greenough et al., 1973; Uylings et al., 1978; Connor et al., 1982; Turner and Greenough, 1985; Kempermann et al., 1997; Leggio et al., 2005; Jung and Herms, 2012). Unsurprisingly, the changes in dendritic morphology are accompanied by synaptic alterations, with EE resulting in increased numbers of synapses and synaptic contacts (Jones et al., 1997; Briones et al., 2004; Landers et al., 2011), which could enhance cortical synaptic transmission and hence, alter cortical excitation/inhibition balances.

One particularly notable EE-induced change is neurogenesis, which may contribute to enhanced cognitive performance (Kempermann et al., 1997, 1998; Nilsson et al., 1999; Bruel-Jungerman et al., 2005). Cell proliferation, improved neuronal survival and functional integration of new neurons have all been demonstrated to occur in the adult dentate gyrus after EE (Kempermann et al., 1997, 1998; Van Praag et al., 2000; Lu et al., 2003; Bruel-Jungerman et al., 2005) and pharmacological inhibition of cell proliferation during EE prevented hippocampal neurogenesis and any improvement in a hippocampal-based memory task (Bruel-Jungerman et al., 2005). Decreased neurogenesis has been linked to cognitive decline (Drapeau et al., 2003, 2007), and restoration of neuronal proliferation and survival leads to an improvement in cognitive behavior (Kempermann, 2002).

The enhanced hippocampal neurogenesis, improved neuronal cell survival, increased synaptic density and dendritic branching (Kempermann et al., 1997, 1998; Bruel-Jungerman et al., 2005), and the growth factor up-regulation that is discussed below, have all been suggested to be responsible for EE-induced improvements in spatial and non-spatial learning and memory and enhanced spatial searching strategies (Van Praag et al., 2000; Schrijver et al., 2002; Nithianantharajah and Hannan, 2006; Kulesskaya et al., 2011; Vedovelli et al., 2011; Leger et al., 2012). Increased habituation to novel objects has also been attributed to a decrease in activation of hippocampal neurons during novel object exposure in enriched animals, in contrast to the increased activation seen in animals housed in standard conditions when exposed to objects in novel environments (Zhu et al., 1997; Leger et al., 2012). These results support the idea that EE-induced morphological changes contribute to alterations in behavior, through changes in overall neuronal function.

It must be noted here that similar neurogenesis has not been demonstrated to occur in cortex after EE, but this may be for a want of study not for an absence of the effect. In the absence of this effect, it is difficult to speculate to what extent neurogenesis contributes to EE-induced changes in cortical neuronal functionality or cortex-based processes. We will argue below that, in any case, neurogenesis is not required to occur in cortex to produce the EE-induced changes in responses and in functionality and that those changes can be produced by alterations in the balance between excitation/inhibition interplay that shapes neuronal responses and function.

### Molecular changes

The EE-induced structural and functional changes described above occur through molecular cascades that involve increases in neurotrophic factor and neurotransmitter levels (Van Praag et al., 2000; Mohammed et al., 2002; Will et al., 2004; Nithianantharajah and Hannan, 2006), and increased expression of regulatory proteins that enhance the number and stability of synapses, increase cell proliferation, and promote neurotransmitter release (Rampon et al., 2000; Frick and Fernandez, 2003; Nithianantharajah et al., 2004).

Neurotrophic factors of particular importance to EE include brain derived neurotrophic factor (BDNF) and nerve growth factor (NGF), with levels of both increasing following exposure to exercise and EE (Pham et al., 1999; Birch et al., 2013) in brain regions including cortex, hippocampus and cerebellum (Torasdotter et al., 1998; Angelucci et al., 2009). In the adult brain, BDNF and NGF promote experience-dependent plasticity by enhancing synaptic plasticity, signaling, learning and memory (Kang and Schuman, 1995; Torasdotter et al., 1998; Pham et al., 1999; Bekinschtein et al., 2011). Increases in neurotrophins appear to underlie improved motor and cognitive function after EE (Falkenberg et al., 1992; Henriksson et al., 1992; Linnarsson et al., 1997; Bekinschtein et al., 2011; Gelfo et al., 2011; Bechara and Kelly, 2013; Birch et al., 2013) since suppression of BDNF levels causes deficits in neurogenesis, learning behavior and memory (Linnarsson et al., 1997; Minichiello et al., 1999; Rossi et al., 2006; Heldt et al., 2007; Furini et al., 2010).

Similarly, neurotransmitter levels are also affected by exposure to EE, suggesting a role in the mediation of brain plasticity. Serotonin is important in promoting neuroplasticity (Maya Vetencourt et al., 2008; Baroncelli et al., 2010a), and EE induces an increase in serotonin receptor expression and in serotonin levels (Rasmuson et al., 1998; Koh et al., 2007; Baroncelli et al., 2010a). Levels of other neurotransmitters associated with synaptic plasticity, such as acetylcholine and noradrenaline, also increase following EE (Por et al., 1982; Galani et al., 2007; Brenes et al., 2009). Increased cortical responsiveness may then be attributed to increases in synaptic transmission efficacy and synaptic strength (Mainardi et al., 2010) brought about by EE-induced molecular changes.

In conjunction with these changes in neurotransmitter levels, excitatory activity also shifts following EE housing, with increases in hippocampal extracellular glutamate levels coupled with an enhanced expression of AMPA and NMDA receptors (Tang et al., 2001; Naka et al., 2005; Segovia et al., 2006). Changes in hippocampal field potentials have been attributed to factors that include increased AMPA receptor-mediated transmitter binding, increased expression of AMPA and NMDA receptor subunits, increased dendritic spine density and upregulation of growth factors (Sharp et al., 1985; Green and Greenough, 1986; Foster and Dumas, 2001; Eckert and Abraham, 2010). There is also evidence of synaptic plasticity, in the form of increased dentate gyrus LTP, in the hippocampus after physical activity, which is an important component of the EE experience (Van Praag et al., 1999). The role of these changes in excitation will be discussed in greater detail below where we argue that a principal mechanism through which EE alters brain function and behavior is by promoting a shift toward excitation in neuronal responses.

### Role of changes in cortical excitation/inhibition balance in EE-induced changes in neuronal functionality

As shown above, EE-induced brain plasticity is likely to occur through the combination of structural and biochemical changes that can impact on neuronal functionality. We believe that there is substantive evidence that one particularly important end-effect through which EE alters neuronal function is alterations in the balance between excitation and inhibition in cortex (Engineer et al., 2004; Percaccio et al., 2005, 2007). This E/I balance is a critical factor in regulating cortical neuronal functionality and critical periods of cortical plasticity which occur throughout development are governed by this E/I balance (Hensch and Fagiolini, 2005; Levelt and Hubener, 2012) whereby immaturity of cortical inhibition promotes plasticity while maturation of inhibitory circuits results in the decrease in plasticity associated with cortical maturation (Huang et al., 1999; Fagiolini and Hensch, 2000). Shifts in this E/I balance may also play a major role in the experience-dependent cortical plasticity, which includes plasticity induced by EE or deprivation, that occurs outside developmental critical periods (Hensch and Fagiolini, 2005; Sale et al., 2007; Benali et al., 2008; Maya Vetencourt et al., 2008; Megevand et al., 2009; Baroncelli et al., 2010b, 2011; Luz and Shamir, 2012; Maya-Vetencourt et al., 2012). Changes in neuronal function in the adult brain suggest that EE exposure causes a reactivation of forms of neuronal plasticity generally seen only in the developing, immature brain (Chang and Merzenich, 2003; Chang et al., 2005) in which inhibitory mechanisms are immature.

Experience-dependent changes in response strength and sensitivity in adult sensory cortices have been attributed to decreased levels of cortical inhibition (Buonomano and Merzenich, 1998; Baroncelli et al., 2011), which shift cortical E/I ratios to favor excitation. Studies have demonstrated the importance of GABA synthesis in promoting plasticity (Hensch et al., 1998; Harauzov et al., 2010) after MD, and disruption of GABA-ergic inhibition through pharmacological treatments or EE reinstates plasticity to restore OD plasticity in adult visual cortex (Sale et al., 2007; Maya Vetencourt et al., 2008; Harauzov et al., 2010; Zhou et al., 2011; Maya-Vetencourt et al., 2012). Similarly, Zhou et al. (2011) found that EE-induced plasticity in auditory cortex was accompanied by a decrease in GABA receptor subunit expression. Sale et al. (2007) have also shown that EE exposure results in a decrease in basal extracellular GABA levels in the visual cortex, and that EE-induced plasticity can be countered by pharmacologically increasing inhibitory activity. EE-induced decreases in cortical inhibition have been demonstrated in studies of auditory and visual cortex, through decreases in GABA receptor subunit expression, inhibitory synapse density and basal levels of GABA (Beaulieu and Colonnier, 1987; Zhou et al., 2011; Jakkamsetti et al., 2012), while GAD67 expression has also been shown to decrease following exposure to EE (Scali et al., 2012; Tognini et al., 2012).

However, decreased inhibition may just be one mechanism underlying EE-induced plasticity via shifts in the excitation/inhibition balance, with studies also suggesting an increase in cortical excitation with EE. Nichols et al. (2007) have shown that exposure to EE induces an AMPA-receptor mediated increase in EPSC amplitudes in supragranular layers of the auditory cortex, with no changes in GABA-ergic transmission, while the increase in PPD after EE demonstrated by Percaccio et al. (2005) suggests an increase in the transmitter release probability of excitatory synapses.

Taken together, the results presented in this section suggest that EE exerts its effects through molecular changes, which in turn support changes in morphology and neuronal function. These results also suggest that in conditions such as brain injury, which result in abnormal neuronal activity, EE-induced plasticity may have the potential as a therapy to steer neuronal activity toward a more functionally relevant state. This is particularly the case when it is considered that excitation/inhibition shifts may also occur in brain injury (Ding et al., 2008; Alwis et al., 2012; Johnstone et al., 2013).

## EE and the damaged brain

Considering that the brain plasticity evoked by EE results in various behavioral benefits, it is hardly surprising that EE (and specifically the form we have termed “generic” EE) has been proposed as a putative therapy for neurological conditions ranging from Alzheimer's disease through to ischemia/stroke (Faherty et al., 2005; Jadavji et al., 2006; Buchhold et al., 2007; Nithianantharajah et al., 2008; Wang et al., 2008; Hu et al., 2010; Valero et al., 2011; Du et al., 2012). Indeed, EE ameliorates the behavioral and pathological deficits associated with many of these conditions: for example, in models of Alzheimer's disease, EE enhances neuronal proliferation, survival and maturation, leading to improved cognition (Hu et al., 2010; Valero et al., 2011), while in studies of stroke/ischemia, EE improves sensorimotor function, such as impaired gait and limb placement (Buchhold et al., 2007; Wang et al., 2008).

Our focus in this review is on the potential role of EE as a therapy for TBI, based on its known effects on brain changes induced by TBI, especially in the sensory cortices. For this consideration, it is necessary to first define some of the basic features of TBI and its effects on brain and behavior. There are two major forms of TBI—focal and diffuse. Focal brain injury is caused by direct, localized damage to the brain, while diffuse injury is most commonly caused by indirect forces, such as during rapid acceleration/deceleration of the head (Andriessen et al., 2010; Alwis et al., 2013). TBI affects approximately 2 million individuals every year in the US alone (Faul et al., 2010) and has been shown to result in a number of persistent sensory deficits, which are thought to underlie TBI-associated cognitive disabilities (Caeyenberghs et al., 2009; Davis and Dean, 2010; Lew et al., 2010; Folmer et al., 2011). People with mild to moderate diffuse TBI usually recover motor skills fully, but have other prolonged deficits, including cognitive deficits and memory loss, likely from axonal injury (Strich, 1956; Adams et al., 1999; Graham et al., 2000; Little et al., 2010).

In TBI there are often substantial and prolonged functional deficits in cognition, memory and movement (Gagnon et al., 1998; Draper and Ponsford, 2008; Park et al., 2008; Faul et al., 2010; Risdall and Menon, 2011) and these are invariably viewed as resulting from damage to brain areas specific to those functions. What has been consistently overlooked is that most TBI sufferers show deficits in how they process sensory information. What we see, hear, touch is used to understand the world and guide complex behaviors like thinking, movement, or memory; sensory processing deficits easily affect these behaviors. It has been recognized that at least some impairments may involve disruption of the integration of sensory input (Brosseau-Lachaine et al., 2008; Patel et al., 2011). In humans, speeded motor tasks and response time tasks are also affected in mild/moderate TBI (Bawden et al., 1985; Haaland et al., 1994), and animal studies have shown persistent abnormal sensory behavior (McNamara et al., 2010), again suggesting disturbances in sensorimotor processing, and there are many long-lasting sensory and cognitive impairments even after motor function has recovered (Narayan et al., 2002; Draper and Ponsford, 2009; Faul et al., 2010; Risdall and Menon, 2011).

Consistent with this hypothesis of a sensory cortices basis for persistent cognitive, memory and motor deficits in TBI, experimental TBI causes significant time-dependent changes in neuronal excitability in sensory cortices (Hall and Lifshitz, 2010; Ding et al., 2011; Alwis et al., 2012). In the immediate post-TBI period, changes in neuronal activity occur across all cortical layers, and consist in a depth-dependent (from the cortical surface) suppression of responses to all types of simple and complex naturalistic stimuli (Johnstone et al., 2013; Yan et al., 2013). However, by the long-term (8–10 weeks post-TBI), effects are found only in the upper cortical layers, layers 2 and upper layer 3, and decrease with cortical depth such that there are no long-term changes in input layer 4 (Alwis et al., 2012); further the changes in the upper layers are no longer a suppression of responses but, rather, a hyper-excitation (Alwis et al., 2012). These persistent effects are consistent with an imbalance in cortical excitation/inhibition (Ding et al., 2011; Alwis et al., 2012).

Given the known effects of EE on the E/I balance in cortex, we believe that there is potential for EE to remediate TBI-induced changes in neuronal function by restoring the cortical excitation/inhibition balance, to improve sensorimotor and cognitive behavior. However, there is no current literature probing the effects of EE specifically on neuronal function post-TBI, highlighting the need for studies examining the mechanisms underlying the EE-induced functional improvements that have been reported in the literature. Hence, in the following section of the review, we will first consolidate and discuss studies that have examined the effects of EE after experimental brain injury, which have focussed on mainly behavioral effects. We will then discuss possible mechanisms through which EE acts to improve functional outcomes post-injury, based on the known mechanisms underlying the effects of EE (as discussed above). Finally, we will review the implementation and efficacy of EE as a therapeutic option to remediate brain injury in a clinical setting.

## The beneficial effects of EE post-TBI

Only a few studies have investigated the effects of EE on functional recovery post-experimental TBI and shown that exposure to EE ameliorates motor and cognitive deficits and TBI-induced histopathologies (Hamm et al., 1996; Passineau et al., 2001; Hicks et al., 2002; Sozda et al., 2010; De Witt et al., 2011; Matter et al., 2011; Monaco et al., 2013; Bondi et al., 2014), with some very limited work on the effect of EE on TBI-induced sensory morbidities. These studies have predominantly studied the use of generic EE in the treatment of TBI, except for some work that has included the use of multi-modal sensory stimulation with generic EE (discussed further below).

EE improves TBI-induced cellular histopathologies: EE-treated animals show decreases in lesion volume, increased neuronal survival, and reduced neuronal degeneration in cortex (Passineau et al., 2001; Lippert-Gruner et al., 2007; Monaco et al., 2013). Muthuraju et al. (2012) recently reported an EE-mediated decrease in cell death, in conjunction with an increase in neurogenesis in the striatum, post-TBI. Additionally, in correlation with the positive effects on motor and cognitive function, decreased apoptosis and lesion volume have also been shown using a combination of multi-modal sensory stimulation and EE (Maegele et al., 2005a; Lippert-Gruner et al., 2007). These effects suggest that EE may be able to ameliorate or attenuate damage caused by secondary injury processes, which are often complex and dynamic in nature.

The effects of EE on motor and cognitive function after TBI have only been investigated in TBI models that cause a mixture of focal and diffuse TBI (Hamm et al., 1996; Passineau et al., 2001; Hicks et al., 2002; Hoffman et al., 2008; Sozda et al., 2010; De Witt et al., 2011; Matter et al., 2011; Monaco et al., 2013), and to date, no studies have examined the effect of EE after a purely diffuse model of TBI. The studies of EE effects in mixed model TBI have demonstrated positive effects of EE on neuromotor and cognitive function, especially for spatial navigation and memory. Hamm et al. (1996) found that at 11–15 days after mixed-model TBI, animals exposed to generic EE displayed elevated spatial memory function in the Morris Water Maze (MWM) test, when compared with TBI animals housed in standard conditions. EE housing also improved cognitive functioning to levels comparable to those of sham controls (Hamm et al., 1996). These findings are similar to reports demonstrating EE-induced recovery of spatial navigation and spatial memory after hippocampal/cortical lesions (Will et al., 1977; Einon et al., 1980; Whishaw et al., 1984). EE-induced improvements in spatial navigation, increases in spatial acquisition task rate, as well as improved spatial memory, have been demonstrated in other experimental models of mixed-model TBI (Passineau et al., 2001; Hicks et al., 2002; Wagner et al., 2002; De Witt et al., 2011; Matter et al., 2011; Monaco et al., 2013). EE-mediated recovery of locomotor activity and motor function in beam-walking and rotatod tasks has also been documented post-TBI (Wagner et al., 2002; De Witt et al., 2011; Matter et al., 2011; Monaco et al., 2013), while EE exposure also improved recovery time of forelimb function after CCI (controlled cortical impact) injury (Smith et al., 2007).

While EE on its own has all of these benefits for motor and cognitive function, there is also evidence that the use of additional multi-modal sensorimotor stimulation together with EE can improve cognitive and motor function (Maegele et al., 2005b; Lippert-Gruener et al., 2007; Lippert-Gruner et al., 2007, 2011) at both acute (7 and 15d; Maegele et al., 2005a,b; Lippert-Gruner et al., 2007), and chronic (30d; Lippert-Gruener et al., 2007) time-points post-injury. Such enhanced stimulation is thought to better mimic rehabilitation paradigms used in clinical settings in the treatment of brain injury. Maegele et al. (2005a) have demonstrated that enhanced sensory stimulation in combination with EE improves behavioral outcomes more than the use of EE on its own (Maegele et al., 2005a), suggesting that any neuroplasticity conferred by increased stimulation may be therapeutically relevant.

Only a very few studies have examined the efficacy of EE in ameliorating sensory deficits after TBI, with only one study (Johnson et al., 2013) reporting that EE exposure completely recovers TBI-induced sensory neglect, a condition where there is a reduction in responsiveness to sensorimotor stimuli (Kim et al., 1999). Conversely, in a unilateral cortical lesion model of brain injury, Rose et al. (1987) found that EE did not facilitate recovery from sensory neglect post-lesion. These contradictory results may be explained by differences in the nature of injury, or even the timing of EE exposure: in the Johnson et al. (2013) study, 15 days of EE exposure occurred immediately *prior* to TBI, whilst Rose et al. (1987) examined the effects of 6 weeks of EE exposure commencing 10–12 days post-lesion. We will demonstrate below that the timing of the application of EE is absolutely critical for ameliorating TBI-induced behavior deficits.

In most of these studies, the type of EE applied has been what we have termed “generic” EE. Whether fortuitous or planned, this form of EE, which must engage a range of sensory, motor, cognitive and social behaviors, appears to greatly improve recovery post-TBI. Thus, Hoffman et al. (2008) indicate that EE-induced functional recovery may depend on task-specific experience: post-TBI, animals show enhanced recovery of motor function (e.g., beam traversing and balancing) and spatial learning and memory (decreased latency to locate a platform in the MWM), when exposed to both EE *and* task-specific training for the motor and cognitive tests. They also suggest that the enhanced motor, social and cognitive stimulation provided by housing in EE conditions could, in themselves, contribute to the improved motor and cognitive they observed. It is possible that exposure to general EE vs. specific EE dictates the level of functionality that is conferred; although it is true that often studies using specific EE focus on tasks related to the aspect of EE that they enhance. Considering the ambiguity that remains concerning this issue, further investigations are required to determine whether exposure to task-specific experience or specific EE can improve overall functionality in a range of tasks.

It has been suggested that improved sensorimotor function after brain injury may actually be attributed to behavioral compensation rather than functional recovery; improvements on multi-sensory tasks, such as a MWM test, could be due to the use of cues from alternate (presumably undamaged) modalities (Finger, 1978; Rose et al., 1987, 1993; Kolb et al., 1996). This view receives support from studies that show that EE has much more limited or negligible benefit in tasks involving a single sensory modality (Rose et al., 1987, 1988). However, there is also no reason why the two effects could not o-exist and in keeping with this possibility, studies have shown a degree of EE-induced functional recovery after cortical injury, accompanied by an observed difference in the movements of the animals during task performance such as skilled reaching post-injury (Whishaw et al., 1991; Rowntree and Kolb, 1997; Kolb, 1999), suggesting that perhaps both recovery and compensation may account for improved functional outcomes after injury, possibly through the recruitment of uninjured cortical circuits (Kolb, 1999). We therefore turn now to a consideration of the potential mechanisms whereby EE could improve outcomes after TBI.

## Mechanisms promoting improved behavioral and pathological recovery

To understand how EE could promote recovery after TBI, it is necessary to understand some of the mechanisms underlying TBI. This is not a simple endeavor since neurodegeneration caused by TBI occurs as a result of a number of complex and dynamic inter-related mechanisms (Smith et al., 1991; Hicks et al., 1993; Pierce et al., 1998; Hall and Lifshitz, 2010; McNamara et al., 2010). Only a few studies have examined how EE impacts on these complex molecular and anatomical factors affected in TBI or any other form of brain injury. Thus, ideas of how EE might produce improvements in sensorimotor and cognitive behavior after TBI or any brain injury are often based on extrapolations of the known actions of EE in the normal brain.

In uninjured animals, EE enhances neurogenesis, improves neuronal survival, and decreases apoptotic cell death (Kempermann et al., 1997; Van Praag et al., 2000; Lu et al., 2003; Bruel-Jungerman et al., 2005), all of which have been linked to improved behavioral recovery after TBI (Passineau et al., 2001; Gaulke et al., 2005; Sozda et al., 2010; Monaco et al., 2013). Additionally, Miller et al. (2013) have also recently demonstrated a decrease in hippocampal volume loss in TBI patients, with increased engagement with cognitively, physically and socially demanding activities, supporting the role of EE in directly preventing neuronal death.

Post-injury EE housing has been shown to decrease injury-induced lesion volume, increase synaptic density, increase post-synaptic density (PSD) thickness, increase dendritic spine density and dendritic branching in supragranular and infragranular layers of injured cortex (Biernaskie and Corbett, 2001; Ip et al., 2002; Johansson and Belichenko, 2002; Xu et al., 2009a,b). Such structural changes may lead to EE-enabled compensation and recovery of function after injury (Rose et al., 1987; Kolb and Gibb, 1991; Whishaw et al., 1991; Passineau et al., 2001; Will et al., 2004). Under uninjured conditions, EE-induced structural changes are thought to occur due to upregulation of trophic factors such as VEGF and BDNF which promote cell survival and plasticity, and an increase in activation of transcription factors of proteins mediating plasticity (Young et al., 1999; Rampon et al., 2000; Keyvani et al., 2004; Will et al., 2004; Gaulke et al., 2005; Hoffman et al., 2008; Sozda et al., 2010; Monaco et al., 2013; Ortuzar et al., 2013). Interestingly, studies have reported an increase in BDNF expression after TBI (Hicks et al., 1997; Chen et al., 2005), with no further increase following exposure to EE (Chen et al., 2005) suggesting that EE-induced benefits for recovery after TBI may not depend on increasing the levels of trophic factors.

The effects of TBI may also be exerted through inflammatory processes: as we have noted previously (Alwis et al., 2013), activation of inflammatory cascades as part of the normal cellular response to injury can cause further injury to the already damaged brain (Menge et al., 2001; Morganti-Kossmann et al., 2002). The inflammatory response in TBI involves production of pro-inflammatory cytokines like interleukin-1 (IL-1), IL-6 and tumor necrosis factor (TNF-a), and anti-inflammatory cytokines such as IL-10 and IL-12, all of which are seen in the cerebrospinal fluid of TBI patients within a few hours of the primary injury. Inflammatory cytokines IL-1a, IL-1b, and IL-18 are also increased after TBI (Menge et al., 2001; Morganti-Kossmann et al., 2002). Interestingly, EE is able to decrease levels of pro-inflammatory molecules such as tumor necrosis factor alpha (TNF-α) and interleukin 1b (IL-1b; Briones et al., 2013) in the cortex and hippocampus post-injury. Given the up-regulation of these factors by TBI, the EE effect may attenuate secondary-injury related damage.

EE also induces an increase in neurotransmitter levels such as noradrenaline and dopamine, NMDA receptor expression, and brain metabolic activity, all of which are altered in TBI and have been implicated in impairment of motor and cognitive function; thus, exposure to EE could possibly regulate TBI-induced changes in these factors (Brenner et al., 1983; Boyeson and Feeney, 1990; Liljequist et al., 1993; Dietrich et al., 1994; Hamm et al., 1996).

We noted above that some work indicates that EE in animal models of epilepsy attenuates onset of seizures, a functional consequence of aberrant neuronal excitability after TBI (Pitkanen and McIntosh, 2006; Hunt et al., 2013; Shultz et al., 2013). While the exact mechanisms underlying this protective effect are unknown, it is thought to occur through an EE-induced enhancement of trophic support, changes in receptor expression and enhanced neurogenesis (as previously described; Liu et al., 1993; Cheng et al., 1995; Young et al., 1999; Reibel et al., 2000; Korbey et al., 2008).

This review shows that overall, there are insufficient data available to decide if EE effects on all of the TBI-induced molecular events noted above are all (or any of them) involved in the beneficial effects of EE in TBI. Given the limited amount of knowledge of the effects of EE specifically in TBI, it is worth also considering how EE acts therapeutically in other neurological disorders. The general trend of how EE has benefits in other brain disorders is summarized in Table 1, where it can be seen that EE acts to increase neurogenesis, dendritic branching and spine density, and increase the expression of growth factors (Johansson and Ohlsson, 1996; Young et al., 1999; Jadavji et al., 2006; Pereira et al., 2007; Gelfo et al., 2011; Valero et al., 2011). Similarly, recent work by Koopmans et al. (2012) has also demonstrated increased spinal cord progenitor cell differentiation and increased serotonergic innervation after experimental SCI, while others have shown increased dendritic spine density in the motor cortex (Kim et al., 2008) and increased BDNF levels (Berrocal et al., 2007). These studies, as well as those focussing on TBI, show that EE appears to be a promising post-injury treatment to improve sensorimotor and cognitive function in brain injury, most likely due to similar underlying structural and molecular mechanisms. Due to the wide and inter-connected nature of the effects of EE, it is likely that a combination of both molecular and morphological changes needs to occur in order to see improvements in neuronal function and behavior.

**Table 1:** **Behavioral, morphological, and molecular effects of EE in various neurological disease conditions**.

**Neurological disorder**	**Behavioral effects**	**Morphological effects**	**Molecular effects**	**References**
Stroke/hypoxia-ischemia	Improved motor function (reaching, beam-walk, rotating pole)	Increased dendritic length and branching in cortex	Increased growth factor expression	Ohlsson and Johansson, 1995; Johansson and Ohlsson, 1996; Biernaskie and Corbett, 2001; Johansson and Belichenko, 2002; Risedal et al., 2002; Dahlqvist et al., 2004; Gobbo and O'Mara, 2004; Komitova et al., 2006; Buchhold et al., 2007; Pereira et al., 2007; Rojas et al., 2013
	Improved declarative memory (Novel object recognition test)	Preserved dendritic spine density loss/increased spine density in hippocampus, cortex		
	Improved spatial learning and memory (Morris water maze, Radial arm maze)	Decreased cortical infarct volume		
		Enhances cell proliferation		
Lesions	Improved motor function (posture, ladder climb)	Increased dendritic length in cerebellum	Increased growth factor expression	Kelche and Will, 1982; Held et al., 1985; Kolb and Gibb, 1991; Bindu et al., 2007; Frechette et al., 2009; De Bartolo et al., 2011; Gelfo et al., 2011
	Improved spatial learning and memory (Morris water maze)	Increased dendritic branching and spine density in hippocampus, cortex		
Epilepsy	Increased seizure resistance	Decreased hippocampal cell death	Increased growth factor expression	Young et al., 1999; Auvergne et al., 2002; Faverjon et al., 2002; Rutten et al., 2002; Koh et al., 2007; Korbey et al., 2008
	Increased exploratory activity (Open field)	Increased neurogenesis	Enhanced expression of neuronal and synaptic plasticity mediators	
	Improved spatial learning (Morris water maze)			
Huntington's disease	Delayed onset of motor deficits	Delays degenerative loss of cerebral volume	Increased growth factor expression	Van Dellen et al., 2000; Hockly et al., 2002; Spires et al., 2004; Lazic et al., 2006; Nithianantharajah et al., 2008; Wood et al., 2010
	Improved spatial memory (Barnes maze, Morris water maze)	Attenuates deficits in hippocampal neurogenesis	Increased synaptic protein expression	
		Reduced aggregation of huntingtin protein fragments		
Alzheimer's disease	Improved spatial learning and memory (Morris water maze, Barnes maze)	Increased/decreased Aβ and amyloid deposition	Increased growth factor expression	Jankowsky et al., 2003, 2005; Levi et al., 2003; Arendash et al., 2004; Wen et al., 2004; Lazarov et al., 2005; Berardi et al., 2007; Cracchiolo et al., 2007; Levi and Michaelson, 2007; Valero et al., 2011
	Improved working memory (Radial arm water maze)	Increased neuronal progenitor cell proliferation	Increased synaptophysin expression	
		Increased/decreased neurogenesis Decreased progenitor cell survival			
Parkinson's disease	Improved motor function (skilled reaching task)	Decreased dopaminergic neuron and transporter loss	Increased growth factor expression	Bezard et al., 2003; Faherty et al., 2005; Jadavji et al., 2006
		Decreased cell death		

*Summary of findings from studies that have investigated the effects of EE after various neurological conditions*.

## Timing of the use of EE as a therapy after TBI

**Figure 2:**
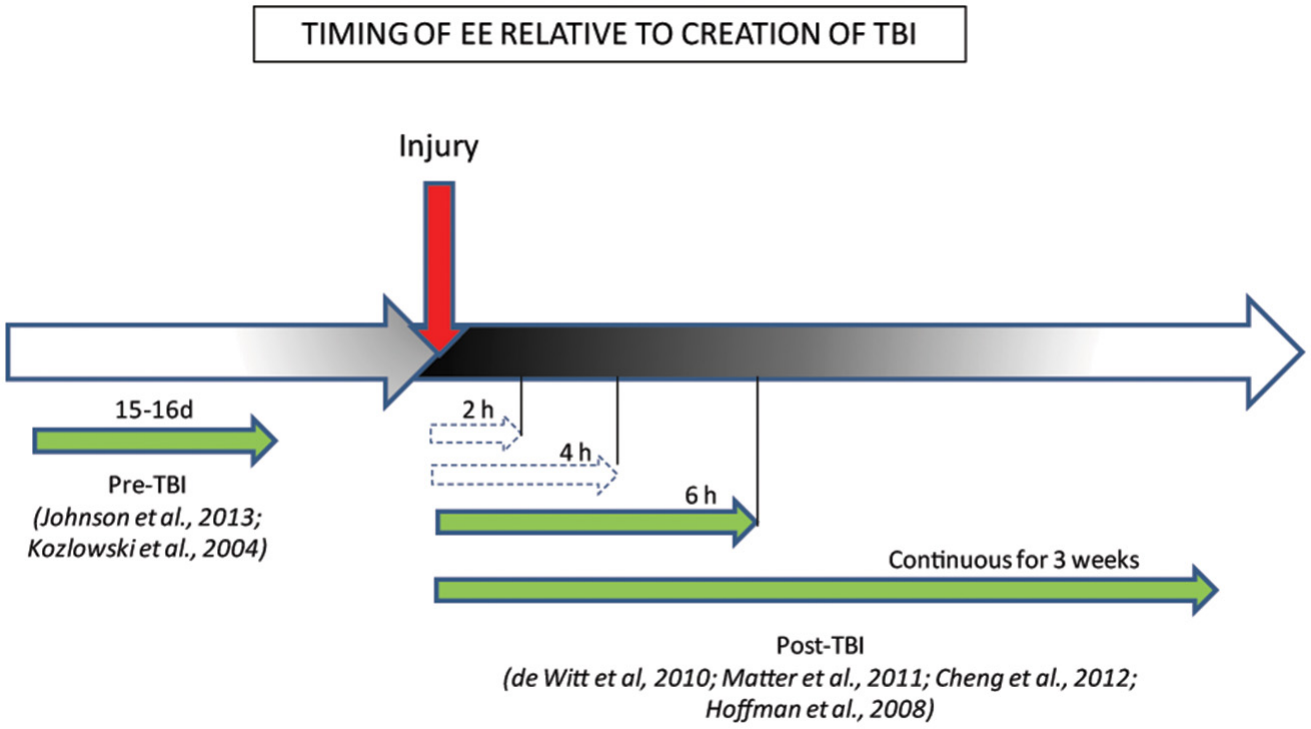
**Behavioral benefits conferred by the timing/duration of EE relative to creation of TBI**. The efficacy of EE treatment when administered pre/post-injury is represented by green full-line arrows which indicate EE timing conditions that ameliorated behaviors, while white dashed arrows indicate EE timing conditions that failed to ameliorate behaviors.

Some clues as to the potential mechanism by which EE could rescue brain function in TBI comes from the finding that the timing and duration of EE are important factors governing motor and cognitive recovery post-TBI (Figure 2; Hoffman et al., 2008; De Witt et al., 2011; Matter et al., 2011; Cheng et al., 2012). Thus, Hoffman et al. (2008) reported that recovery of motor and cognitive function, such as beam-walking and spatial learning and memory, depended on an optimal time and length of EE exposure relative to time after injury. After mixed focal-diffuse TBI, even a short period of EE exposure (6 h) was sufficient to improve motor and cognitive behavior to a level comparable to the enhanced performance seen after much longer (3 weeks), continuous exposure to EE (Hoffman et al., 2008; De Witt et al., 2011; Matter et al., 2011). The benefits of EE were not dose-dependent, however, as task performance in animals exposed to shorter periods of EE (2 and 4 h) did not differ significantly from those of injured animals housed in standard conditions, suggesting a minimal threshold of EE exposure is needed for beneficial effects (De Witt et al., 2011). EE-induced plasticity has persisting effects such that even limited exposure (3 weeks) to EE post-TBI can result in long-term protection from memory deficits, as assessed by the MWM task, for up to 6 months after animals are withdrawn from EE conditions (Cheng et al., 2012), making it an ideal candidate for therapy post-TBI.

Recent studies have also demonstrated that even pre-injury exposure to EE is neuroprotective (Kozlowski et al., 2004; Johnson et al., 2013). Kozlowski et al. (2004) showed that brief (15d) EE exposure in immature rats at p21, prior to TBI in adulthood (approximately 3 months old), results in a paradoxical increase in cortical lesion volume, but coupled with faster recovery of forelimb function, assessed by foot fault and asymmetry tests. Similarly, exposure to 15 days of EE immediately pre-TBI attenuates spatial and long-term memory deficits in the MWM task, in a manner similar to that seen after post-injury EE exposure (Johnson et al., 2013). However, the relevance of such a model in a clinical setting is limited. It is however, likely that, in accord with the Hebbian theory of plasticity, pre-injury exposure to EE acts mainly to strengthen existing connections in the uninjured brain, which may carry forward post-injury. This is likely to occur through the upregulation of trophic factors such as BDNF, which are shown to increase after exposure to motor enrichment before and after injury (Kleim et al., 2003). In contrast, post-injury EE exposure acts mainly to develop and strengthen new/previously silent/remaining connections to compensate for the damage in existing pathways (Taub et al., 2002), while elevated growth factor expression acts to limit the spread of damage (Kleim et al., 2003). This would suggest that although pre- and post-injury EE-exposure is beneficial for protection and/or recovery from injury, the underlying mechanisms may be different.

The fact that even pre-injury exposure to EE can ameliorate the effects of TBI may be taken to indicate that the benefits of EE are independent of the TBI-induced molecular and structural changes that cause deficits to brain and behavior. However, this is, as yet, an unsafe assumption for the reasons that brain processes are so highly inter-linked and often use common pathways. For example, as noted above, TBI increases inflammatory cascades while EE reduces many of the same cascades; thus down-regulation of these cascades by pre-exposure to EE may reduce subsequent TBI-induced activation of the inflammatory mechanisms and thereby reduce secondary injury processes, producing neuroprotection.

## Beyond the bench

The current body of literature on the effects of EE in neurological disease indicates that EE represents significant therapeutic potential, on its own and in combination with pharmacological treatments (Kline et al., 2007, 2010, 2012), by inducing neuroprotective mechanisms involving molecular, structural, and functional processes to improve histopathologies and behavioral outcomes. Considering the many positive effects of EE demonstrated after experimental brain injury, it would be logical to try to aid recovery by applying EE in a clinical setting. Certainly, intellectually, physically and socially active lifestyles (that are akin to EE) have been linked to improved cognitive function and lower incidences of cognitive impairment, particularly in older, uninjured adults (Seeman et al., 2001; Scarmeas and Stern, 2003; Wilson et al., 2003; Newson and Kemps, 2005; Fujiwara et al., 2009; Voss et al., 2011). Similarly, cognitive enrichment early in life has also been linked to improved cognitive abilities in later life (Milgram et al., 2006), while Kramer et al. (2004) have suggested that enhanced cognitive enrichment results in improved crystallized intelligence.

Indeed, the use of EE as a rehabilitative treatment for humans post-TBI has been suggested to be effective in positively influencing long-term outcomes. However, the concept of EE for humans is more complex to define than what constitutes as EE for animals, as factors such as engagement and motivation play a role in classifying the level of enrichment an individual receives. In a clinical setting, EE can be broadly classified as a paradigm that specifically enhances and promotes engagement with cognitive, social and physical stimulation. An important caveat to the discussion about the role of EE in the treatment of TBI is that post-TBI rehabilitation programs are widely considered to be comparable to enriched environments, in that these programs often comprise of multiple components that are considered hallmarks of EE, which include physical and cognitive therapy, multi-modal stimulation, novelty, duration, functional relevance, and social integration. It has to be noted, however, that specific skill rehabilitation paradigms often do not result in improved general performance in the post-discharge environment, and instead act to improve task-specific performance (Sohlberg et al., 2000; Park and Ingles, 2001). It has instead been suggested that a more generalized treatment would be beneficial in improving overall function (Toglia, 1991; Toglia et al., 2010). Rehabilitation paradigms treating brain injury are often implemented in the acute stages post-injury, in an in-patient hospital setting. Rehabilitation based on EE principles in TBI patients results in better general functional outcomes, such as improved cognitive and motor skills (Willer et al., 1999; Powell et al., 2002; Cifu et al., 2003; Boman et al., 2004; Hayden et al., 2013), and better community integration (Zhu et al., 2001; Cicerone et al., 2004). A number of studies have also shown that increasing the duration and intensity of exposure to rehabilitative therapy results in improved recovery times (Blackerby, 1990; Spivack et al., 1992; Shiel et al., 2001; Zhu et al., 2001, 2007; Slade et al., 2002; Cifu et al., 2003; Cicerone et al., 2004).

It has also been suggested that a lack or an absence of EE is linked to cognitive decline post-injury (Till et al., 2008; Frasca et al., 2013), demonstrating the importance of continued exposure to EE in the post-discharge stages after brain injury. In that sense, a number of factors could contribute to the provision of an appropriate level of enrichment once a patient has left an intensive rehabilitative environment. These factors include ease of access to activities and resources that are cognitively, physically and socially stimulating, as well as support that encourages participation and integration with these environments (Frasca et al., 2013). Frasca et al. (2013) have also suggested that although patients eventually return to an environment that could be considered enriched post-TBI, interactions with these environments may be restricted due to limitations in cognitive and/or physical deficits. This is especially relevant during the transition from the in-patient rehabilitation environment, to post-discharge home environments, where complexity of, and engagement with environments may reduce. A reduction in enrichment in the post-acute period could be detrimental to recovery as studies have shown that functions gained during stimulation of neural pathways (such as during rehabilitation) can be lost through under-use (Rubinov et al., 2009; Warraich and Kleim, 2010; Frasca et al., 2013). Post-discharge, the major forms of therapy mapped onto EE principles include community-based and home-based rehabilitation, with the aim that these programs would aid in improving behaviors and skills required for everyday functioning, improving community integration, and preventing cognitive decline (Fryer and Haffey, 1987; Frasca et al., 2013). Studies have shown that continued enrichment in the form of cognitive rehabilitation in the post-discharge setting (i.e., domestic or vocational environments) increases neuropsychological function, learning and memory (Willer et al., 1999; Boman et al., 2004).

Given the complexity and ethics of manipulations of the environment in humans recovering from TBI, in addition to the difficulties in accurately comparing the effectiveness of various rehabilitation paradigms, questions of the correlation between these effects and EE-induced functional changes remain. Injury heterogeneity also raises challenges in defining exactly what level of enrichment is optimal and beneficial. However, the findings presented in this section strongly suggest that EE or EE-based therapy tailored to the patient's needs could significantly improve outcomes when applied in both the in-patient, acute and post-discharge, chronic settings.

## Conclusion

The studies described here well support the use of EE as a therapeutic paradigm in the treatment of TBI. However, while the results of these studies all show promise in improving TBI-induced histopathologies and sensorimotor and cognitive deficits, there is still much work to be done to clarify our understanding of how EE exerts its effects in disease conditions. Paramount to the understanding of how EE improves behavioral outcomes after injury is the investigation of how EE changes neuronal function post-TBI, of which we know nothing. Only once these effects are unveiled will we be able to implement EE as a treatment option post-injury at its maximum potential.

It is also worth adding the caution that while EE holds promise in its application as a therapeutic tool after brain injury in humans, the complex nature of utilizing EE in a clinical setting makes it difficult to standardize treatment and compare outcomes. It is also true that EE as an experimental paradigm in animal studies has yet to be standardized, with housing conditions, environmental stimuli, number of animals per cage, age of animals at onset of enrichment, as well as the duration of enrichment, varying markedly between studies. The extent of contribution of these factors is particularly relevant when considering the demonstrated neuroprotective effects of EE in neurological disease states, where little is known about how improved functional outcomes relate to changes in neuronal function. Using experimental models that are easily controlled and manipulated, however, would provide us with valuable insight into the therapeutic potential of EE, both in laboratory and clinical settings.

### Conflict of interest statement

The authors declare that the research was conducted in the absence of any commercial or financial relationships that could be construed as a potential conflict of interest.
